# Outer Membrane Vesicle Biosynthesis in *Salmonella*: Is There More to Gram-Negative Bacteria?

**DOI:** 10.1128/mBio.01282-16

**Published:** 2016-08-16

**Authors:** Joachim Reidl

**Affiliations:** Institute of Molecular Biosciences, University of Graz, Graz, Austria

## Abstract

Recent research has focused on the biological role of outer membrane vesicles (OMVs), which are derived from the outer membranes (OMs) of Gram-negative bacteria, and their potential exploitation as therapeutics. OMVs have been characterized in many ways and functions. Until recently, research focused on hypothetical and empirical models that addressed the molecular mechanisms of OMV biogenesis, such as vesicles bulging from the OM in various ways. The recently reported study by Elhenawy et al. (mBio 7:e00940-16, 2016, http://dx.doi.org/10.1128/mBio.00940-16) provided further insights into OMV biogenesis of *Salmonella enterica* serovar Typhimurium. That study showed that deacylation of lipopolysaccharides (LPS) influences the level of OMV production and, furthermore, determines a sorting of high versus low acylated LPS in OMs and OMVs, respectively. Interestingly, deacylation may inversely correlate with other LPS modifications, suggesting some synergy toward optimized host resistance via best OM compositions for *S*. Typhimurium.

## COMMENTARY

Little attention was paid to bacterium-derived membrane vesicles (MVs) until the last few decades. As we know now, outer membrane vesicles (OMVs) are subcellular, spherical, bilayered, protein-decorated vesicles of a size of 20 to 250 nm in diameter and have similar lipid compositions as the outer membranes (OMs) of their donor cells. MVs are widely recognized to be released by bacteria, eukaryotes, and archaea and seem to serve for interkingdom communications (1, 2). Current research on OMVs includes host-pathogen interactions, potential therapeutic uses, and mechanisms of OMV biosynthesis. OMVs appear to have many functions, including the spread of virulence factors into the host cells and influence on the host defense, e.g., triggering immune responses (3). The role of OMVs in interspecies interactions in bacterial communities is also a matter of study, since antibacterial factors can be incorporated as cargo. For example, proteins such as β-lactamase and murein hydrolase are contained in OMVs derived from *Pseudomonas aeruginosa* and function in rescuing or destroying “friends or foes” (4). Additional studies have furthered a potential role for OMVs in interspecies communication by identifying quorum-sensing molecules in *P. aeruginosa* OMVs and also DNA in enterohemorrhagic *Escherichia coli* strain O157:H7 OMVs, which were further shown to mediate horizontal gene transfer (4). In the field of vaccine development, numerous bacterial pathogens are the focus for OMV-derived vaccines, since OMVs provide immunomodulatory and adjuvant-like activities and display many naturally occurring microorganism-associated molecular patterns (3, 5, 6). Ultimately, research focused on OMV protein enrichment (2) will prove important for future tailoring of OMVs to modulate their synthesis, alter membrane compositions, and spike the membranes with proteins of interest for biotechnological, immunological, or therapeutic applications (3, 7). However, with all that in mind, we still lack a basic understanding of how OMVs are synthesized naturally.

Nearly 35 years ago, Wensink and Witholt (8) were some of the first scientists to report on OMVs derived from growing *Escherichia coli* cells, and they indicated an imbalance of lipoproteins in OMVs versus OMs. Based on that finding, they proposed a sort of bulging model for OMV biogenesis which relied on distinct spatially limited synthesis of peptidoglycan, in comparison to OM production, which correlates with an absent lipoprotein-mediated OM connectivity. Since then, more scientists have tried to define an OMV biogenesis pathway, with ideas such as accumulation of peptidoglycan fragments or misfolded proteins in the periplasmic space, or enrichment of membrane curvature-inducing molecules initiating OMV production (2, 9). Current research activities are focused on the molecular basis of OMV biosynthesis, including, for example, secretion and sorting of lipids into the OMVs or OMs (10, 11).

The recent study by Elhenawy et al. (12) contributes significantly to our understanding of OMV biogenesis by showing that mainly deacylated LPS are found in OMVs. This implies that some sorting mechanisms exist to distinguish LPS molecules based on the content of acyl residues contained on the GlucNAc backbone of the lipid A moiety. Furthermore, those authors proposed that lipid remodeling correlates with OMV formation, suggesting that OMV bulging and budding is a consequence of the accumulation of deacylated LPS species in the OM. For their studies, they selected *Salmonella enterica* serovar Typhimurium, a well-known pathogen of the intestine that causes an immense burden of infectious disease in humans. *S*. Typhimurium has the hallmark ability to alter its OM, e.g., due to modification of the lipid A by adding aminoarabinose and phosphoethanolamine and by other modifications of the membrane lipid content; these modifications increase the diffusion barrier efficiency of the OM to resist and increase bacterial survival in the face of immune responses (13). Evidence in the field suggests that OMVs or MVs are released even during intracellular growth, as shown by Elhenawy et al. and also recently for *Mycobacterium tuberculosis* (14). Elhenawy et al. analyzed the lipid A content of the OMs and OMVs in a *pagL* mutant which lacked the gene for a PhoPQ-regulated lipid A lipase enzyme and which showed no difference from the wild type in its LPS profile. However, when comparing cells overexpressing a PagL_inactive_ mutant versus a PagL_active_ strain, they observed an overall increase of OMV production for the latter. Analysis of the lipid A contents of OMVs and the OMs of the PagL_active_ clone revealed that significantly more deacylated lipid A was found in the OMVs, based on mass spectrometry analysis. They further characterized the *in vivo* growth of *S*. Typhimurium propagating in a J774A.1 macrophage cell culture and compared the OMV level produced by a *pagL* knockout mutant versus that of the wild type. The results showed that OMVs can be detected in the cytosol of macrophages, and the amount of wild-type-derived OMVs represented a 4-fold increase over that of the *pagL* mutant. The authors suggested a possible scenario whereby hexa-acylated lipid A, not modified by other substitutes (e.g., by aminoarabinose or phosphoethanolamine), might be deacylated to a penta-acylated form. Subsequently, such a form is then allocated to OMVs, thereby providing space in the OM for fully modified LPS molecules, which in turn establish a high barrier function to increase persistence and resistance against host attack. In summary, in the study of Elhenawy et al., novel original findings were represented which clearly expose new amplitudes for the process of OMV biogenesis in *Salmonella*. Expectations will be high to explore this as a common strategy for Gram-negative bacteria.

Previous research has made progress toward our understanding of the biogenesis of the OMs of Gram-negative bacteria, specifically regarding LPS biogenesis (15). Since the OM is the origin of OMVs, mechanisms of both biogenesis pathways may be linked. It was recently reported that the Mla transporter system, which is involved in maintenance of lipid asymmetry between the OM and inner membrane (11), is also connected to the biogenesis of OMVs (10). Repression or deletion of Mla pathway components positively influences OMV production, based on phospholipids accumulation in the OM. In this context, it seems possible that the lipid contents of the LPS molecules also have an influence on OMV biosynthesis, as shown by the results of Elhenawy et al. Since PagL, or its orthologs, and the Mla pathway are widely distributed among Gram-negative bacteria, a more general picture of OMV biogenesis is becoming evident. Characterizing conditions that test both pathways will reveal whether a physiologically relevant synergy is unleashed for OMV biosynthesis.
